# Tight junction physiology of pleural mesothelium

**DOI:** 10.3389/fphys.2014.00221

**Published:** 2014-06-24

**Authors:** Alexander G. Markov, Salah Amasheh

**Affiliations:** ^1^Department of General Physiology, St. Petersburg State UniversitySt. Petersburg, Russia; ^2^Department of Veterinary Medicine, Institute of Veterinary Physiology, Freie Universität BerlinBerlin, Germany

**Keywords:** pleura, tissue barrier, mesothelial cells, tight junctions, claudins

## Abstract

Pleura consists of visceral and parietal cell layers, producing a fluid, which is necessary for lubrication of the pleural space. Function of both mesothelial cell layers is necessary for the regulation of a constant pleural fluid volume and composition to facilitate lung movement during breathing. Recent studies have demonstrated that pleural mesothelial cells show a distinct expression pattern of tight junction proteins which are known to ubiquitously determine paracellular permeability. Most tight junction proteins provide a sealing function to epithelia, but some have been shown to have a paracellular channel function or ambiguous properties. Here we provide an in-depth review of the current knowledge concerning specific functional contribution of these proteins determining transport and barrier function of pleural mesothelium.

## Introduction

**Figure 1:**
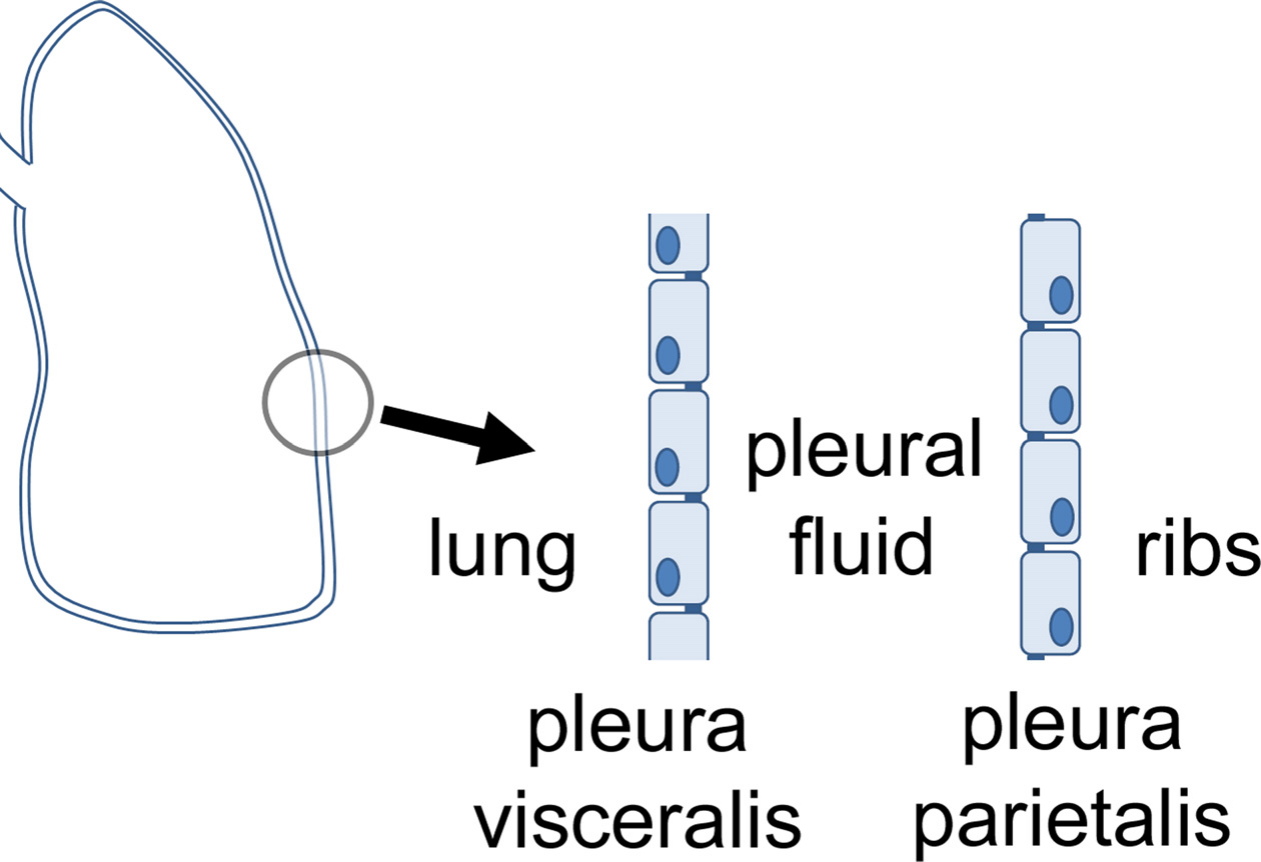
**Orientation of visceral and parietal pleura mesothelium**.

Pleura, peritoneum and pericardium are squamous epithelia of mesothelial origin, which line lungs, chest cavity, abdomen, and heart, respectively. Pleura can be divided in the pleura visceralis or pleura pulmonalis covering lungs, and pleura parietalis covering the ribs. Both are delimitated by a single layer of epithelial cells which attach to a lamina propria, and have different functions regarding transport and barrier properties. Between the two layers, the pleura cavity is located (Figure 1). The pleura provides a fluid layer for lung movement, and therefore is necessary for breathing This small volume of pleural liquid is a result of filtration and absorptive processes, and its volume and composition is tightly regulated (Zocchi, 2002). An imbalance of this regulation can result in an accumulation of fluid between the parietal and the visceral pleura, a condition defined as pleural effusion (Light, 1997).

The difference between hydrostatic and colloid-osmotic pressure of pleural liquid and capillary blood results in filtration of pleural liquid in the pleural cavity. In caudal parietal pleura, the elimination of liquid from a pleural cavity occurs through lymphatic vessels. In this context, further mechanisms related to mesothelial cell activity have been discussed to be more relevant, including a solute-coupled absorption of liquid from the pleural space (Zocchi, 2002). However, vectorial transport across epithelia depends on a concerted action of transcellular transport mechanisms in combination with paracellular channel and barrier functions, and several transcellular transport mechanisms have been identified to participate in the formation of pleural liquid. For analysis, electrophysiological measurements have been performed in different species including man (Hatzoglou et al., 2001; Sarkos et al., 2002; Kouritas et al., 2008; Markov et al., 2011). Moreover, the molecular correlate of paracellular channel and barrier function has been discovered in recent years, namely the tight junction (TJ) which shows a tissue specific expression of tight junction proteins determining the functional properties of the tissues (for review, see e.g., Amasheh et al., 2011; Rosenthal et al., 2012). Neighboring epithelial cells are linked together by these structures, which show a distinct strand pattern in freeze fracture electron micrographs (Staehelin, 1973). Typically tight junctions are located between the apical and the basolateral membrane of epithelial cells, providing not only a *gate* function describing the role for paracellular permeability, but also a *fence* function which is a prerequisite for polarity of the cells, as it delimits diffusion of basolateral and apically located integral membrane molecules to the opposite membrane compartment, respectively (Tsukita et al., 2001). Taken together, both transcellular and paracellular determinants of transport and barrier function can be regarded to contribute to the generation of different ionic composition of pleural fluid compared to plasma (Table 1).

**Table 1:** **Comparison of ionic composition of pleural fluid and plasma**.

**Ion**	**Pleural fluid**	**Plasma**	**Species**	**References**
Na^+^	139 (141^*^)	142 (151^*^)	Rabbit, anesthesia	Zocchi et al., 1991
	141	149	Rats, no anesthesia	Rolf and Travis, 1973
K^+^	4.48	4.42	Rabbit, anesthesia	Zocchi et al., 1991
Cl^−^	96 (98^*^)	93 (102^*^)	Rabbit anesthesia	Zocchi et al., 1991
	111	122	Rats, no anesthesia	Rolf and Travis, 1973
	109	113	Rabbit, no anesthesia	Sahn et al., 1979
HCO^−^_3_	29	26	Rabbit anesthesia	Zocchi et al., 1991

* *Corrected according to the concentration of electrolytes per liter of serum water or of pleural liquid water*.

Furthermore, inflammation was identified as one major mechanism perturbing barrier integrity, as shown previously in inflammatory bowel diseases (Amasheh et al., 2009a). In this context, also an effect of pleura inflammation on tight junction proteins was observed (Markov et al., 2011).

## Transport and barrier mechanisms in pleura mesothelium

### Electrophysiological properties of pleura mesothelium

In Ussing chambers, transmesothelial potential (*V_m_*), and transmesothelial resistance (*R_m_*) have been reported. The experiments revealed differences concerning absolute values, which might be attributed to different specimens and preparative protocols. However, these differences were rather marginal and in comparison with studies focusing on established epithelial models of kidney and intestine (Amasheh et al., 2002; Markov et al., 2010), these values indicated rather leaky properties of both, visceral and parietal pleura (Table 2).

**Table 2:** **Comparison of transmesothelial potential (*V_m_*) and resistance (*R_m_*) values measured in different species including human tissue specimens**.

**Species**	**Visceral pleura**		**Parietal pleura**		**References**
	***V_m_* (mV)**	***R_m_* (Ω ·cm^2^)**	***V_m_* (mV)**	***R_m_* (Ω ·cm^2^)**	
Canine	0.06	20	0.03	22	Payne et al., 1988
Sheep	0.4	22	0.5	22	Hatzoglou et al., 2001
	–	21	–	20	Zarogiannis et al., 2006
	–	–	0.5	19 (costal)	Zarogiannis et al., 2007
	–	–	0.6	21 (diaphragmal)	Vogiatzidis et al., 2006
	–	–	–	38	Sarkos et al., 2002
Human	–	–	–	26	Sarkos et al., 2002
				21 (Cranial, mediastinal) 18 (Caudal)	Kouritas et al., 2008
	1.6	14	1.3	18	Markov et al., 2011

*– not provided*.

**Figure 2:**
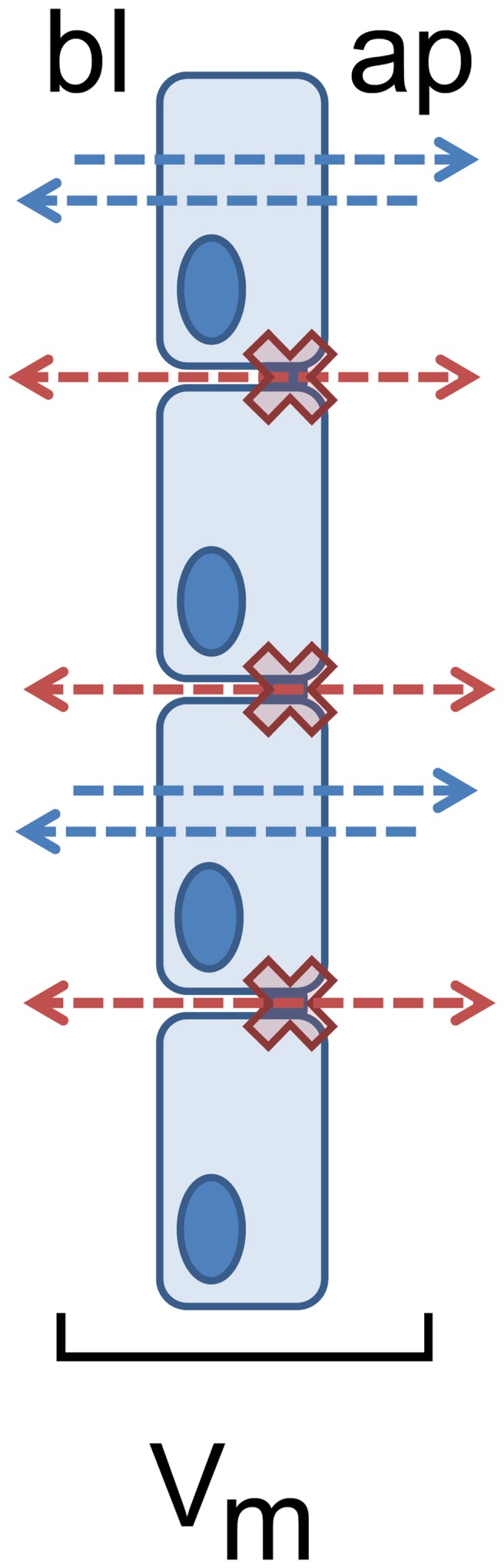
**Restricted paracellular permeability as a prerequisite for pleural transmesothelial potential**. Transport routes are indicated as blue (transcellular) and red (paracellular) arrows between apical (ap) and basolateral (bl) compartments. The paracellular barrier is indicated as red crosses.

The existence of electrophysiological parameters indicates transport and barrier mechanisms, as transmesothelial transport implies a transmesothelial barrier function to limit the paracellular pathway during active transport mechanisms, restricting back leak and therefore allowing vectorial transport and the generation of gradients, as e.g., also demonstrated in human gastrointestinal tract before (Amasheh et al., 2009b). This functional interplay of transport and barrier function can be identified by transmesothelial potential measurements; Figure 2.

There are specific regional functional differences in organs with large epithelial surface, as shown in detail for intestine and kidney (Markov et al., 2010; Amasheh et al., 2011). In intestine, this segment specific correlation of barrier properties was analyzed by a combination of electrophysiological and molecular analyses (Markov et al., 2010). In analogy the segmentation of different pleura areas may also be associated with differential barrier properties. Electrophysiological experiments employing paracellular flux markers of different masses, and protein expression profiles allow a detailed analysis of these variations. Currently, different electrophysiological properties are known concerning parts of parietal pleura, namely mediastinal, costal and diaphragmal pleura. In sheep, transmesothelial resistance of costal pleura showed a lower transmesothelial resistance than diaphragmal pleura (Zarogiannis et al., 2007) whereas in human pleura, a higher resistance of cranial pleura compared to caudal pleura was reported (Kouritas et al., 2008; Table 2).

For analyses of pleural transport function, ouabain and amiloride have been employed. Whereas ouabain is a selective blocker of the—typically basolaterally located—Na^+^/K^+^-ATPase, application of amiloride inhibits the epithelial sodium channel (ENaC), typically located in the apical membrane. Application of ouabain and amiloride had different effects on human parietal pleura. Whereas in the caudal region, an increase of transmesothelial resistance was reported, in the cranial and mediastinal pleura no significant ouabain effect was observed (Kouritas et al., 2008). This apparent contradiction might indicate that there is no generally uniform mapping, as some studies refer to the cranial-caudal axis, and others referring to single rib areas (Kouritas et al., 2008).

### Pleural transmesothelial transport systems

Transport properties have been analyzed in pleura in detail by inducing a hydrothorax (Agostoni and Zocchi, 1990). This model includes an injection of Ringer solution into the pleural cavity to replace single ions, and the application of a variety of blockers including ouabain and amiloride. With this technique, a solute-coupled absorption of liquid from the pleural space was observed (Agostoni and Zocchi, 1990; Zocchi et al., 1991). Moreover, a general epithelial phenotype was identified, which included the expression of the Na^+^/K^+^-ATPase. Interestingly, the distinct localization of the Na^+^/K^+^-ATPase is not resolved yet: Western blots revealed an expression of the Na^+^/K^+^-ATPase a1 subunit in both, visceral and parietal pleura of rabbits, and mesothelial primary cell culture of rabbits (Sironi et al., 2007, 2008). Existence of this subunit is characteristic for epithelia and endothelium (Krenek et al., 2006; Gupta et al., 2012). Employing the hydrothorax model, injection of Ringer's solution with the Na^+^/K^+^-ATPase blocker ouabain resulted in a reduction of water absorption, and a decrease of ion absorption from the pleural cavity (Agostoni and Zocchi, 1990; Zocchi et al., 1991). These findings indicated that the Na^+^/K^+^-ATPase was located in the apical membrane of pleural mesothelium, but a discrimination between visceral and parietal pleura was approached only in later experiments employing the Ussing chamber technique. In these studies, analysis of transmesothelial resistance of different pleural specimens with ouabain revealed differences of Na^+^/K^+^ ATPase localization in visceral and parietal pleura (Hatzoglou et al., 2001), as ouabain induced an increase of transmesothelial resistance only in visceral pleura when added to the apical compartment, whereas in parietal pleura, an effect of ouabain on both, the apical and basolateral side of the epithelium, was observed.

Further evidence for the localization of Na^+^/K^+^-ATPase of parietal pleura was found in human tissue. In Ussing chamber experiments employing parietal pleura specimens, apical addition of ouabain showed an effect (Kouritas et al., 2008).

As until now no immunohistochemical detection of the localization has been published, these results cannot fully provide an evidence for localization of Na^+^/K^+^-ATPase in pleura mesothelium, though. This still makes it difficult to define the transcellular absorption from and secretion into the pleural cavity in detail. Another indicator of typical epithelial properties is provided by apical expression of the epithelial sodium channel (ENaC) and exchangers, which also contribute to vectorial transport. ENaC is the limiting factor for epithelial sodium absorption in a variety of tissues and organs, including the distal nephron and colon epithelium (Amasheh et al., 2000). For proper function in epithelia, functional co-regulation of the tight junction to limit paracellular back leak has been reported (Amasheh et al., 2009b).

Evidence of ENaC in parietal and visceral pleura was provided by the hydrothorax model employing amiloride injection, which decreased water absorption, and transport of Na^+^ and Cl^−^ from the pleural cavity (Agostoni and Zocchi, 1990; Zocchi et al., 1991). Terbutaline, an activator of ENaC, as well as the specific ENaC inhibitor amiloride, were injected in mice pleural cavity, leading to an increase, and decrease of water absorption from the pleural cavity, respectively (Jiang et al., 2003).

Application of amiloride from the apical side of human and sheep parietal pleura increases transmesothelial resistance (Sarkos et al., 2002; Kouritas et al., 2008). In contrast, a different group reported that amiloride from the apical side of parietal pleura did not change transmesothelial resistance, but increased the value when added to the basolateral side (Hatzoglou et al., 2001).

This apparent contradiction could be resolved by Nie et al. (2009); when ENaC was apically detected in primary culture of human mesothelial cells and murine parietal pleura (Nie et al., 2009). Although these studies did not include a mapping of the expression in pleura as shown e.g., on functional level (Kouritas et al., 2008), they might become important for the understanding of pathophysiological regulatory mechanisms of transcellular pleural transport, shown for histamine and prostaglandins, recently (Kouritas et al., 2011, 2012).

In visceral pleura, Ussing chamber studies on sheep tissue specimens did not reveal effects of amiloride, when applied to the apical or basolateral side, indicating no functional ENaC expression at all (Hatzoglou et al., 2001). Therefore, it remains an open question if ENaC is localized in the apical membrane of visceral pleura.

In pleural fluid, concentration of glucose is comparable with blood levels. As glucose is present in the pleural space, a transport mechanism for glucose can be expected. In accordance, Western blotting experiments revealed expression of the sodium-glucose linked transporter-1 (SGLT1) in visceral and parietal pleura of rabbit and sheep, and human primary cells (Sironi et al., 2007, 2008). Functional properties were analyzed by experiments using the SGLT-blocker phloricin in the hydrothorax model, which reduced water absorption from the pleural space (Zocchi et al., 1996). Na^+^-Glucose cotransport can be molecular candidate for solute-coupled absorption of liquid from the pleural space, indicating an epithelial phenotype as also found e.g., in intestine and kidney.

Taken together the analyses of transport function, a clear indication of a typical epithelial phenotype is not given. To discriminate an epithelial phenotype in more detail, a lateral diffusion of molecules has to be restricted by tight junctions, though. As epithelial cells typically show a tissue- and organ-specific expression pattern of claudins, these proteins may provide more insight regarding the epithelial functions of pleura.

## Physiological contribution of tight junction proteins

The ignition of molecular tight junction protein research occurred in 1993, when the group of Shoichiro Tsukita described the first discovery of a molecular correlate of barrier function, namely occludin (Furuse et al., 1993). Before this time point, it was even not clear if the tight junction, which had been visualized in electron microscopic images for decades before, was of protein or lipoid origin. This finding soon catalyzed the establishment of a new research field, resulting in a broad variety of novel basic physiological as well as clinically related findings. Soon after Tsukita's pioneer work in the field of molecular tight junction analysis, a second landmark paper was published, this time presenting the first members of the family of claudins, namely claudin-1 and -2. Paralleled by the finding that occludin might not be the most crucial factor for barrier integrity, as the complete knock out did not show a perturbation of barrier properties (Furuse et al., 1998; Schulzke et al., 2005), a series of further publications highlighted the functional contribution of single claudins to major epithelial functions (Amasheh et al., 2011). These studies led to a classification of single members of the claudin family in barrier building (sealing), pore-forming (paracellular channel function) and intermediate claudins, which still is valid and can be regarded as a general outcome of tight junction research. Finally, a third type of tight junction protein was described, which led to a novel classification of non-claudin tight junction proteins as members of the TAMP family, namely tricellulin, MarvelD3, and occludin (Ikenouchi et al., 2005; Steed et al., 2009).

**Figure 3:**
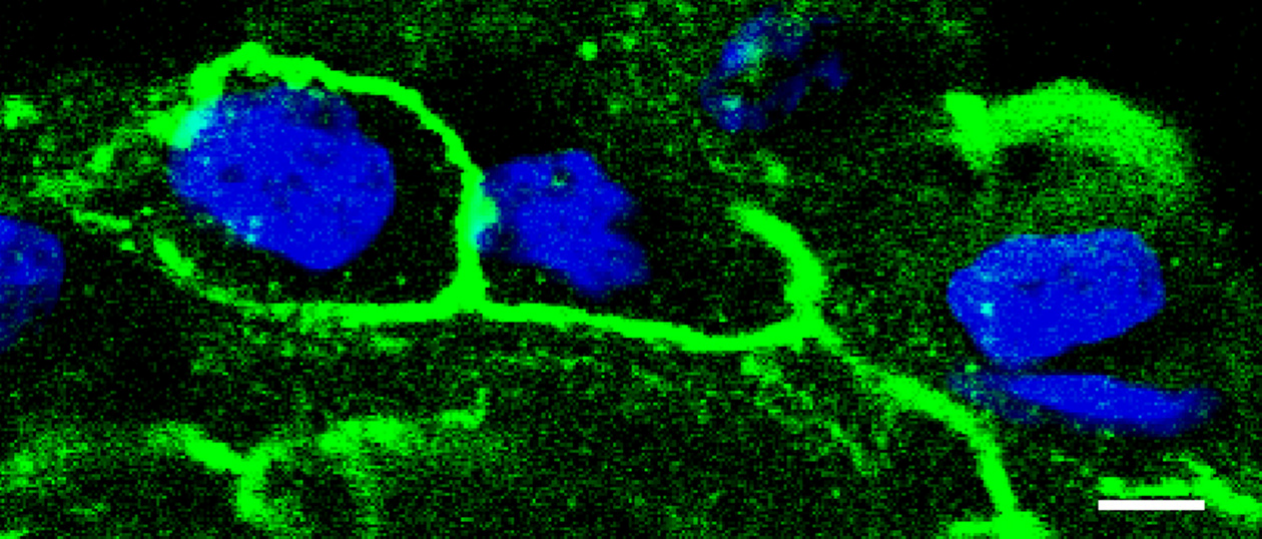
**Detection of tight junction proteins in pleural cells**. Immunostaining of occludin with anti-occludin (green), detected by confocal laser scanning microscopy reveals a honeycomb-like distribution of tight junctions in pleural cells (visceral pleura, typical experiment, nuclei stained in blue, bar: 5 μm).

Among the variety of tetraspan TJ proteins that have been reported to be localized within TJ strands of different organs, occludin (Furuse et al., 1993) and claudins (Furuse et al., 1993) have been identified to primarily provide a barrier against the paracellular passage of ions. Although occludin was the first tetraspan membrane protein that was detected within TJ strands (Furuse et al., 1993), its contribution to barrier properties is still discussed, though (Schulzke et al., 2005). At least, ubiquitous detection in immunofluorescent stainings, established occludin as a general marker of TJ localization, which has been evaluated in many studies (Amasheh et al., 2002, 2005, 2009a,b; Dittmann et al., 2014; Markov et al., 2014). Moreover, occludin can also be visualized in pleura (Figure 3).

In contrast, members of the claudin family have been attributed to the organ-specific properties of epithelia, as e. g. shown in intestine, mammary gland, kidney and lungs (Kaarteenaho et al., 2010; Kirk et al., 2010; Markov et al., 2010, 2012). The single contribution of claudins to barrier properties varies, though. As e.g., claudin-1, -3, and -5, decrease tight junctional ion permeability, other claudins have been demonstrated to specifically mediate paracellular permeability, as it has been shown in detail for claudin-2 which forms a paracellular channel (Amasheh et al., 2002, 2005; Furuse et al., 2002; Milatz et al., 2010).

Information on the molecular correlate of barrier function in the pleura reaches back to the pre-molecular era of tight junction research, when desmosomes and TJs have been discovered as intercellular contacts by electron microscopy (Wang, 1974), and a final breakthrough was achieved by detection of claudins and the correlation with functional barrier properties (Markov et al., 2011).

**Figure 4:**
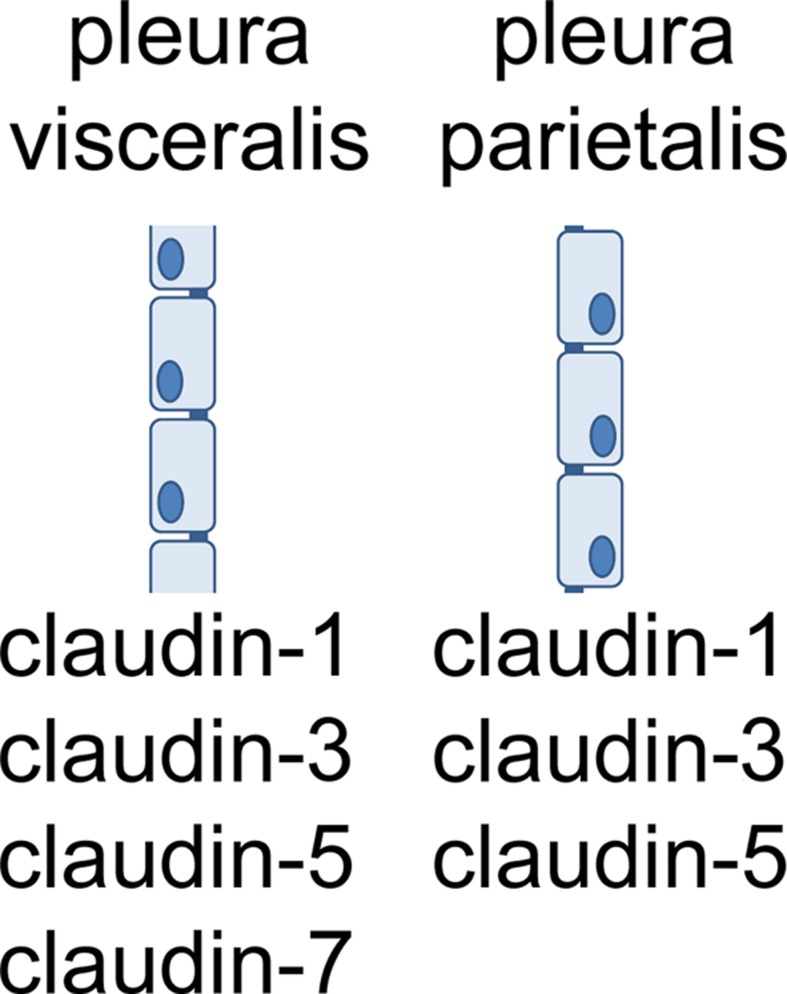
**Claudins of visceral and parietal pleura**.

Recently, for the first time a combined analysis of pleura mesothelial barrier function and expression of TJ proteins was performed. In this study, claudin-1, -3, -5, and -7, were detected in visceral pleura. In parietal pleura, the same TJ proteins were detected, except claudin-7 (Markov et al., 2011; Figure 4). Moreover, in inflamed pleura, claudin-2 was induced, indicating a typical pathophysiological mechanism (see paragraph 4).

According to the functional contribution to barrier properties, claudins can be divided in three groups, namely (i) sealing tight junction proteins, (ii) claudins mediating paracellular permeability and (iii) claudins with ambiguous function. Among the claudins detected in pleura mesothelium, claudin-1, -3, and -5 belong to the first group, whereas claudin-2 belongs to the second, and claudin-7 to the third group.

### Pleura proteins with pore forming, sealing, and ambiguous function

Claudin-1-deficient mice die within hours after birth because of dehydration. These animals show a severe weight loss due to evaporation of water through the skin (Furuse et al., 2002). Therefore, claudin-1 is regarded as one major barrier-building TJ protein.

The functional contribution of claudin-3 has been analyzed in detail by analysis of stably transfected cells, recently (Milatz et al., 2010). This study demonstrates a strong sealing effect on the paracellular pathway regarding the passage of cations, anions, and uncharged solutes. These findings are in accordance with the literature, which reports a ubiquitous presence of claudin-3 in many epithelia including kidney and intestine (Kiuchi-Saishin et al., 2002; Markov et al., 2010).

Sealing properties of claudin-5 have been analyzed in detail in both knock out experiments, and stable transfection of epithelial cells (Nitta et al., 2003; Amasheh et al., 2005). Moreover, recent findings suggest that apart from a tightening of the paracellular barrier against the passage of ions, also the passage of uncharged molecules up to a size of 330 Da is restricted by claudin-5 (Dittmann et al., 2014). Taken together a combination of claudin-1, -3, and -5 can also be found in other epithelia with distinct barrier properties such as the gastrointestinal tract (Markov et al., 2010) and airway epithelium (Coyne et al., 2003).

In contrast to clearly defined claudin properties outlined above, the contribution of claudin-7 to barrier properties is still discussed, as cell type specific differences were observed concerning Cl^−^ and Na^+^ permeability (Alexandre et al., 2005; Hou et al., 2006).

The obtained results indicate that barrier properties of both parietal and visceral pleura mesothelium participate in the formation and determination pleural liquid ionic composition, and different expression levels of occludin, claudin-3, -5, and -7, reflect different extent of functional contributions, respectively.

In contrast to the sealing properties provided by the majority of claudins, claudin-2 has been identified to form a paracellular channel selective for small cations and water (Amasheh et al., 2002; Rosenthal et al., 2010). As a TJ protein mediating paracellular permeability, increased claudin-2 expression is discussed to sustain and aggravate inflammation (Amasheh et al., 2009a,b).

## Pleural tight junctions in health and disease

The expression of claudin-1, -3, -5, and -7 in human pleura indicates an important role of specific barrier properties of the mesothelial cell layers (Markov et al., 2011). These findings are in accordance with previous studies, which have underlined specific contributions of single members of the tight junction protein family of claudins to barrier function of other leaky epithelia such as small intestine and proximal tubule (Markov et al., 2010; Amasheh et al., 2011).

Expression of claudins in pleura mesothelium has been shown to be altered in several pathophysiological conditions. These changes can be found in inflammatory events and in cancer. In previous studies, the paracellular channel claudin-2 has been reported to be induced by tumor necrosis factor α (TNFα), which explains e.g., the pathomechanism of inflammatory bowel diseases (Amasheh et al., 2009a, 2010). The two main forms of inflammatory bowel diseases, namely Crohn's disease and ulcerative colitis, as well as collagenous colitis and pouchitis, all typically show a perturbation of TJ protein expression and localization. This mechanism is regarded to be an important factor for the sustained and aggravated course of the diseases mediated by TNFα (Bürgel et al., 2002; Heller et al., 2005; Zeissig et al., 2007; Amasheh et al., 2009a).

In inflamed pleura, a general reduction of tightening TJ proteins and an increase of permeability mediating TJ proteins was reported, recently (Markov et al., 2011). Claudin-2 was reported to be elevated in inflamed pleura, which has also been shown for Crohn's disease, ulcerative colitis, and pouchitis (Heller et al., 2005; Zeissig et al., 2007; Amasheh et al., 2009a). Moreover, in inflamed pleura, a decrease of occludin, claudin-1, -3, -5, and -7 was detected, which also shows parallels with mechanisms reported in inflammatory bowel diseases (Heller et al., 2005; Zeissig et al., 2007; Amasheh et al., 2009a).

In accordance, pleural exudates showed a marked increase of TNFα levels in a mouse lung inflammation model (Mazzon and Cuzzocrea, 2007). Moreover, TNFα is also elevated in other lung pathologies, e.g., tuberculosis and cancer (Qian et al., 2012).

TNFα at higher levels was reported to induce a down-regulation of the tight junction scaffolding protein zonula occludens protein 1 (ZO-1), and an opening of the paracellular barrier (Ma et al., 2004). As a prerequisite of the pathomechanism of TNFα altering tight junction protein expression in lungs, the TNFα receptor TNFR1 was identified (Mazzon and Cuzzocrea, 2007). If, apart from claudin-2, a number of tightening tight junction proteins are reduced due to a general perturbation of ZO-1, this results in a decrease of barrier properties despite reduction of the paracellular pore claudin-2 (Schneeberger and Lynch, 1992; Fink and Delude, 2005; Jacob et al., 2005), though. Taken together, whereas the increase of claudin-2 in pleural inflammation indicates a separate regulatory pathway which has been reported for inflammatory bowel diseases, the general reduction of tightening proteins in inflamed pleura also points at a mechanism reported in a mouse lung inflammation model, which is not even based on an increase of claudin-2, but a universal decrease of tight junction proteins which may less specifically perturb the epithelial barrier. Both regulatory pathways however appear to be primarily targeted by TNFα.

These alterations in claudin expression are in contrast to findings reported in analyses of pleura cancer and mesothelioma, which show an alteration of claudin-4. In this context, Claudin 4 was identified to be an important marker for carcinoma vs. mesothelioma diagnosis in pleural and peritoneal biopsies and effusions, as it is detected in metastatic tumor cells but not in benign forms of mesotheliomas (Facchetti et al., 2007; Lonardi et al., 2011).

## Conclusions

Transport and barrier function of pleura mesothelium indicate typical epithelial characteristics. Expression of the major sealing tight junction proteins-1, -3, and -5 indicate a physiological role of the mesothelial barrier function for pleural liquid formation. Moreover, the effects observed in inflamed pleura, namely an induction of the paracellular pore claudin-2 and the decrease of tightening tight junction proteins are in accordance with regulatory mechanisms observed in inflamed epithelia, as shown e.g., for inflammatory bowel diseases. These findings may contribute to future therapeutic and preventive approaches regarding mesothelia.

## Author contributions

Alexander G. Markov and Salah Amasheh conceived the review, conducted literature survey and wrote the review.

### Conflict of interest statement

The authors declare that the research was conducted in the absence of any commercial or financial relationships that could be construed as a potential conflict of interest.
